# Steamed Ginger Extract (GGE03) Attenuates Obesity and Improves Metabolic Parameters in Association with AMPK Activation and Lipid Metabolism Regulation in High-Fat Diet-Induced Obese Mice

**DOI:** 10.3390/ijms26188950

**Published:** 2025-09-14

**Authors:** Yean Jung Choi, Jae In Jung, Seungtae Lim, Cha Soon Kim, Dae Won Park, Seong Ho Lee, NaYon Hur, Jaewoo Bae, Jae Kyoung Lee, Eun Ji Kim

**Affiliations:** 1 Department of Food and Nutrition, Sahmyook University, Seoul 01795, Republic of Korea; yjchoi@syu.ac.kr; 2 Institute of Nutritional Physiology and Molecular Nutrition, Sahmyook University, Seoul 01795, Republic of Korea; 3 Industry Coupled Cooperation Center for Bio Healthcare Materials, Hallym University, Chuncheon 24252, Republic of Korea; jungahoo@hallym.ac.kr; 4 Research Institute, SRE Service Co., Ltd., Chuncheon 24232, Republic of Korea; stlim@sres.co.kr; 5 Research Institute, Genencell Co., Ltd., Yongin 16950, Republic of Korea; cskim@genencell.co.kr (C.S.K.); parkdw@genencell.co.kr (D.W.P.); shlee@genencell.co.kr (S.H.L.); 6 3H LABS Research Institute, 3H LABS Co., Ltd., Goyang 10391, Republic of Korea; emily@3h-labs.com (N.H.); jerry@3h-labs.com (J.B.); ljk1200@3h-labs.com (J.K.L.)

**Keywords:** steamed ginger extract, AMPK, lipid metabolism, adipogenesis, obesity, functional food

## Abstract

Steamed ginger ethanolic extract (GGE03) has been shown to exert anti-obesity effects, yet its underlying molecular mechanisms remain unclear. This study investigates the metabolic impact of GGE03 on lipid metabolism, adipogenesis, and energy regulation in a high-fat diet (HFD)-induced obesity model. C57BL/6N mice were fed a control diet, a high-fat diet (HFD), or HFD supplemented with GGE03 (50, 100, or 200 mg/kg/day) for eight weeks. GGE03 significantly reduced body weight gain (HFD: 18.1 ± 0.3 g vs. HFD+GGE03 200 mg/kg/day: 13.4 ± 0.2 g, *p* < 0.05) and fat mass percentage (HFD: significantly higher vs. HFD+GGE03 50, 100, 200 mg/kg/day, *p* < 0.05). Serum glucose levels were decreased from 220.2 ± 8.2 mg/dL (HFD) to 169.6 ± 5.9 mg/dL (HFD+GGE03 200 mg/kg/day, *p* < 0.05), and triglyceride levels were reduced from 82.9 ± 4.2 mg/dL (HFD) to 57.2 ± 2.9 mg/dL (*p* < 0.05). Insulin resistance, as measured by HOMA-IR, was improved by up to 54.9% compared to the HFD (*p* < 0.05). Mechanistically, GGE03 administration increased AMPK phosphorylation (p-AMPK/AMPK ratio significantly elevated by HFD+GGE03 100 and 200 mg/kg/day, *p* < 0.05) and upregulated fatty acid oxidation gene expression (*Cpt-1*), while suppressing lipogenesis-related genes (*Srebp-1c*, *Fas*, and *Acc1*). GGE03 improved obesity-related metabolic disturbances in high-fat diet-induced mice, with beneficial effects associated with AMPK signaling and lipid metabolism. These findings suggest the potential of GGE03 as a functional food ingredient for obesity prevention and management.

## 1. Introduction

Obesity is a major global health concern associated with metabolic disorders such as insulin resistance, dyslipidemia, and cardiovascular disease [1,2,3,4,5]. Despite numerous pharmacological interventions, many available treatments have limited efficacy or undesirable side effects [6], necessitating the exploration of safer, natural alternatives [7]. Ginger (*Zingiber officinale*), a widely consumed functional food [8,9], has demonstrated anti-obesity properties in clinical and preclinical studies [10,11,12,13]. While its weight-reducing effects have been well documented, the underlying molecular mechanisms remain inadequately explored, particularly those related to lipid metabolism and adipogenesis.

Steamed ginger ethanolic extract (GGE03) has recently gained attention due to its enhanced bioactive properties [14,15,16], particularly its high concentration of 6-shogaol, a potent phytochemical with anti-inflammatory and metabolic benefits [17,18,19,20,21,22,23]. A recent randomized, double-blind, placebo-controlled clinical trial by Park et al. [12] demonstrated that steamed ginger ethanolic extract significantly reduces body weight and fat mass in overweight and obese individuals. However, human trials cannot fully elucidate the biochemical and molecular pathways responsible for these effects. Given that metabolic processes such as AMP-activated protein kinase (AMPK) activation, fatty acid oxidation, and lipogenesis regulation are difficult to study in human subjects, preclinical animal models remain indispensable for investigating these mechanisms.

Processing methods such as steaming have been shown to alter the chemical composition and enhance the biological activity of ginger [15,16]. During the steaming process, gingerols—major bioactive components of fresh ginger—are thermally converted into shogaols and other dehydrated derivatives such as 1-dehydro-6-gingerdione, which exhibit stronger antioxidant, anti-inflammatory, and metabolic effects compared to their unprocessed counterparts [17,18,19,20]. Previous studies have reported that steamed ginger exhibits improved efficacy in reducing blood lipid levels, enhancing thermogenesis, and modulating energy metabolism [10,11]. Notably, clinical trials involving steamed ginger extracts have demonstrated significant reductions in body weight and body fat percentage in overweight individuals, suggesting its potential as a functional ingredient for metabolic health management [12]. These enhanced physiological effects are believed to be due, in part, to the enrichment of heat-stable compounds that can more effectively target key regulatory pathways involved in lipid metabolism [13,14].

In this context, the use of a steamed ginger extract (GGE03) is particularly noteworthy, as steaming has been shown to alter the phytochemical profile of ginger, enhancing the concentration of heat-stable bioactives such as 1-dehydro-6-gingerdione. While the physiological benefits of ginger and its major compounds like 6-gingerol and 6-shogaol are well known, the comparative advantage of steamed ginger extracts has not been thoroughly investigated. Previous studies have reported improved metabolic outcomes following the ingestion of steamed ginger, yet few have directly compared different extraction methods or quantified the relative abundance of active constituents. Thus, although steamed ginger extract is presumed to exert effects similar to or stronger than raw ginger, its distinct mechanism and chemical profile warrant specific mechanistic validation. The present study seeks to address this gap by characterizing the metabolic actions of GGE03 while acknowledging the need for future comparative studies and component profiling.

AMPK plays a critical role in maintaining metabolic homeostasis [24]. Numerous natural compounds have been shown to activate AMPK and thereby exert anti-obesity effects. For instance, polyphenols such as resveratrol, catechins, and curcumin, as well as ginsenosides and berberine, have demonstrated AMPK-activating activity in both in vitro and in vivo obesity models [25,26,27,28,29]. These compounds enhance mitochondrial function, increase lipid utilization, and suppress adipogenic gene expression. The growing body of literature on AMPK-targeting phytochemicals highlights the therapeutic relevance of plant-based interventions for metabolic disorders. However, the mechanistic action of steamed ginger extracts—particularly those enriched with 1-dehydro-6-gingerdione—remains underexplored in this context. This study addresses this gap by investigating the role of GGE03 in AMPK activation and its downstream metabolic effects in a high-fat diet-induced obesity model.

In this study, we utilize a high-fat diet-induced obesity model in C57BL/6N mice to investigate the mechanistic basis of GGE03’s anti-obesity effects. Specifically, we assess its impact on lipid metabolism by analyzing AMPK activation, adipocyte morphology, and the expression of key genes involved in lipogenesis and fatty acid oxidation. While weight reduction serves as a secondary validation of efficacy, our primary objective is to delineate the molecular pathways through which GGE03 influences adipose tissue remodeling and energy homeostasis.

By focusing on these mechanisms, our study expands upon prior research and provides critical insights into the metabolic actions of GGE03. These findings contribute to the growing body of evidence supporting the role of natural compounds in obesity management and offer a foundation for future translational research, including dose optimization and clinical applications of GGE03.

## 2. Results

### 2.1. Effect of GGE03 on Body Weight, Weight Gain, and Food Efficiency in HFD-Induced Obese Mice

**Figure 1:**
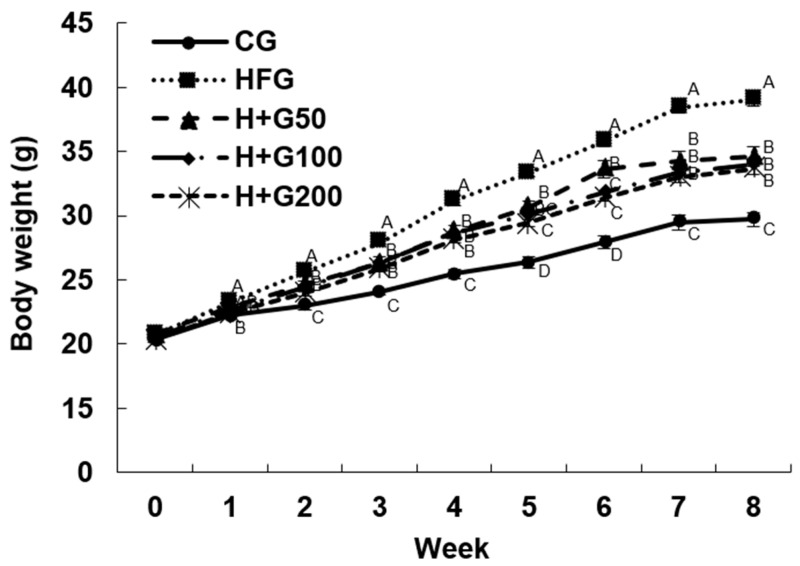
Effect of GGE03 administration on body weight changes over time in high-fat diet (HFD)-fed C57BL/6N mice. The figure illustrates the reduction in body weight of C57BL/6N mice administered with GGE03 at doses of 50, 100, and 200 mg/kg body weight/day for eight weeks. Body weight was recorded weekly for eight weeks, and the x-axis indicates the number of weeks during the experimental period. Mice in the high-fat diet (HFD) group (HFG) exhibited significant weight gain compared to the control diet (CD) group (CG), while GGE03-treated groups showed a dose-dependent reduction in body weight. Data are presented as mean ± standard error of the mean (SEM) (*n* = 10 per group). Different superscript letters indicate statistically significant differences among groups (*p* < 0.05) as determined by one-way ANOVA followed by Duncan’s multiple range test (confirmed with Tukey’s HSD). Groups sharing the same letter are not significantly different. Uncommon abbreviations: GGE03, steamed ginger extract; HFD, high-fat diet; CD, control diet; SEM, standard error of the mean.

In Figure 1, the body weights of the mice were measured weekly over an 8-week period. A significant difference in body weight emerged between the CG and HFG beginning in the first week of treatment, with the GGE03 200 group also showing a significantly lower body weight than the HFG. By the second week, the body weights of all GGE03-treated groups (50, 100, and 200 mg/kg BW) were significantly lower than those of the HFG, with varying degrees of reduction.

In Table 1, the final body weight and body weight gain at the end of the experiment indicate that the HFG had significantly higher weight gain than the CG. After eight weeks, mice in the HFG exhibited a 36.5% increase in body weight compared to the CG. However, weight gain in all three GGE03-treated groups was significantly reduced compared to the HFG, with GGE03 administration at 200 mg/kg resulting in a 25.9% reduction in weight gain relative to the HFG (*p* < 0.05). Daily food intake was significantly lower in the HFG than in the CG. The food efficiency ratio (weight gain/food intake) was significantly higher in the HFG than in the CG. Additionally, the food intake and the food efficiency ratios of all three GGE03-treated groups were significantly lower than that of the HFG, with no significant difference observed among the three GGE03 groups.

### 2.2. Effect of GGE03 on Fat Mass, Adipose Tissue Weight, and Adipocyte Hypertrophy in HFD-Induced Obese Mice

**Figure 2:**
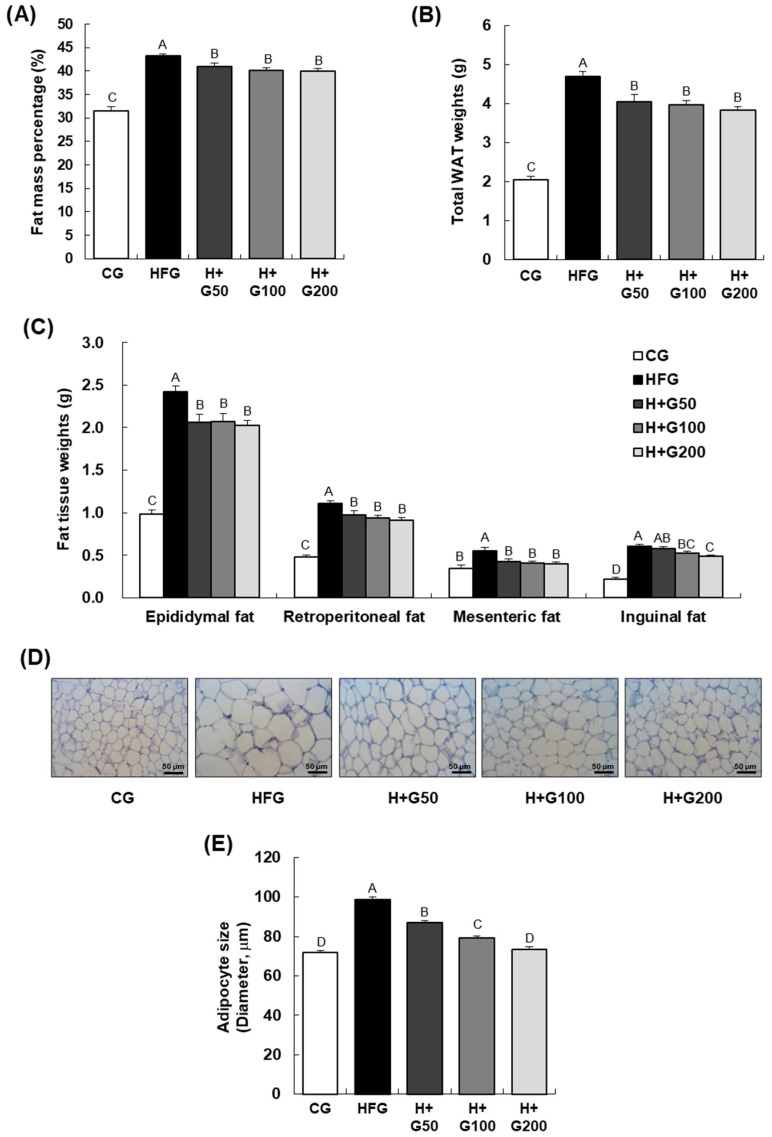
Effect of GGE03 administration on body fat mass percentage, adipose tissue weight, and adipocyte morphology in the epididymal adipose tissue of HFD-fed C57BL/6N mice. This figure demonstrates the impact of GGE03 treatment on body fat distribution and adipocyte morphology in C57BL/6N mice fed a high-fat diet (HFD). GGE03 was administered daily by oral gavage for eight weeks at doses of 50, 100, and 200 mg/kg body weight/day. (**A**) Body fat mass percentage was significantly reduced in GGE03-treated groups compared to the HFD group (HFG), as measured by dual-energy X-ray absorptiometry (DEXA). (**B**) Total white adipose tissue (WAT) weight, including epididymal, retroperitoneal, mesenteric, and inguinal fat weights, was significantly lower in the GGE03-treated groups than in the HFD group (HFG). (**C**) Weights of individual fat depots (epididymal, retroperitoneal, mesenteric, and inguinal) were significantly reduced with GGE03 administration in a dose-dependent manner. (**D**) Representative H&E-stained images show reduced adipocyte size in the epididymal adipose tissue of GGE03-treated groups compared to the HFD group (HFG) (*n* = 5, 200× magnification; scale bar, 50 µm). (**E**) Quantification of adipocyte size reveals significant reductions in the longest diameter of adipocytes in GGE03-treated groups. Quantification of adipocyte size was performed from at least five randomly selected, non-overlapping fields per section, with two sections analyzed per animal (*n* = 10 per group). Data are presented as mean ± standard error of the mean (SEM) (*n* = 10 per group). Statistical significance was determined by one-way ANOVA followed by Duncan’s multiple range test, and key results were confirmed with Tukey’s HSD. Different superscript letters indicate statistically significant differences among groups at *p* < 0.05. Groups sharing the same letter are not significantly different.

As shown in Figure 2A, consistent with the final body weight results in Table 1, the fat mass percentage (%) was significantly higher in the HFG than in the CG, while fat mass in the three GGE03-treated groups decreased compared to the HFG. In Figure 2B,C, both the total weight of white adipose tissue (WAT) and the weights of specific fat depots (epididymal, retroperitoneal, mesenteric, and inguinal) were significantly increased in the HFG. Administration of GGE03 at doses of 50, 100, and 200 mg/kg BW significantly decreased WAT weight in all these areas. Additionally, histological analysis of epididymal adipose tissue was conducted to assess whether GGE03 administration mitigated adipocyte hypertrophy. As shown in Figure 2D, adipocytes in the epididymal adipose tissue of the HFG were larger than those in the CG, while GGE03 administration reduced adipocyte size in this tissue. In Figure 2E, the adipocyte size in the HFG was significantly larger than in the CG, and treatment with GGE03 at 50, 100, and 200 mg/kg BW significantly reduced adipocyte size in the epididymal adipose tissue.

### 2.3. Effect of GGE03 on Serum Glucose, Lipid Profile, and Insulin Resistance in HFD-Induced Obese Mice

In Table 2, serum glucose levels were significantly higher in the HFG compared to the CG, while administration of GGE03 at 100 and 200 mg/kg BW significantly reduced the elevated serum glucose levels induced by the HFG. The HFG also exhibited the highest levels of triglycerides and total cholesterol. Serum triglyceride levels were significantly elevated in the HFG (82.9 ± 4.2 mg/dL), and GGE03 administration at 200 mg/kg reduced triglyceride levels by 31.0% compared to the HFG (*p* < 0.05). Both triglyceride and total cholesterol levels were significantly decreased by GGE03 administration at doses of 100 and 200 mg/kg BW. Insulin levels were elevated in the HFG compared to the CG and were significantly lowered in all three GGE03-treated groups. Regarding insulin resistance, HOMA-IR values increased markedly in the HFG compared to the CG. However, administration of GGE03 at 50, 100, and 200 mg/kg body weight significantly reduced HOMA-IR, with treatment at 200 mg/kg leading to a 54.9% reduction compared to the HFD group, indicating a substantial improvement in insulin sensitivity (*p* < 0.05). Regarding insulin sensitivity, the QUICKI was significantly lower in the HFG group than in the CG; however, administration of GGE03 at 100 and 200 mg/kg BW significantly increased the QUICKI compared to the HFG.

### 2.4. Effect of GGE03 on Serum Leptin and Adiponectin Levels in HFD-Induced Obese Mice

As shown in Table 2, the HFG exhibited higher serum leptin levels and lower serum adiponectin levels compared to the CG. Administration of GGE03 significantly decreased serum leptin levels. However, there was no significant difference in serum adiponectin levels between the HFG and any of the three GGE03-treated groups.

### 2.5. Molecular Effects of GGE03 on Adipogenesis and Lipid Metabolism Gene Expression in HFD-Induced Obesity

**Figure 3:**
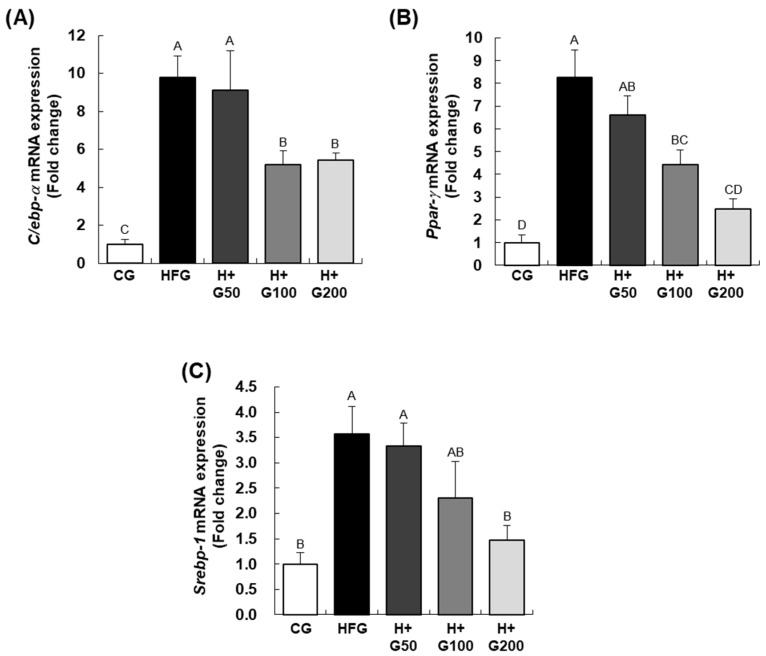
Effect of GGE03 administration on the expression of adipogenic transcription factors in the epididymal adipose tissue of HFD-fed C57BL/6N mice. This figure illustrates the downregulation of adipogenic transcription factors in epididymal adipose tissue following GGE03 treatment in high-fat diet (HFD)-fed C57BL/6N mice. GGE03 was administered daily by oral gavage for eight weeks at doses of 50, 100, and 200 mg/kg body weight/day. Total RNA from epididymal adipose tissue was extracted, reverse-transcribed, and analyzed using real-time PCR. (**A**) The mRNA expression of CCAAT/enhancer binding protein-α (C/ebp-α) was significantly reduced in GGE03-treated groups compared to the HFD group (HFG). (**B**) Peroxisome proliferator-activated receptor-γ (Ppar-γ) expression showed a dose-dependent reduction in GGE03-treated groups. (**C**) Sterol regulatory element-binding protein-1c (Srebp-1c) expression was also significantly reduced in GGE03-treated groups. These results indicate that GGE03 suppresses the expression of key transcription factors involved in adipogenesis. Data are presented as mean ± standard error of the mean (SEM) (*n* = 5 per group). Statistical significance was determined by one-way ANOVA followed by Duncan’s multiple range test, and key results were confirmed with Tukey’s HSD. Different superscript letters indicate statistically significant differences among groups at *p* < 0.05. Groups sharing the same letter are not significantly different.

As shown in Figure 3, to explore the anti-obesity effect of GGE03 at the molecular level, we analyzed the expression levels of CCAAT/enhancer binding protein-α (*C/ebp-α*), peroxisome proliferator-activated receptor-γ (*Ppar-γ*), and sterol regulatory element binding protein-1c (*Srebp-1c*) in epididymal adipose tissue. The HFG demonstrated a significant increase in the hepatic mRNA expression of *C/ebp-α*, *Ppar-γ*, and *Srebp-1c* compared to the CG. In particular, *Srebp-1c* expression was markedly upregulated in the HFD group, while GGE03 administration at 200 mg/kg decreased *Srebp-1c* mRNA levels by 47.2% relative to HFD-fed mice (*p* < 0.05). Expression levels of all three markers were downregulated in a dose-dependent manner across all GGE03-treated groups.

**Figure 4:**
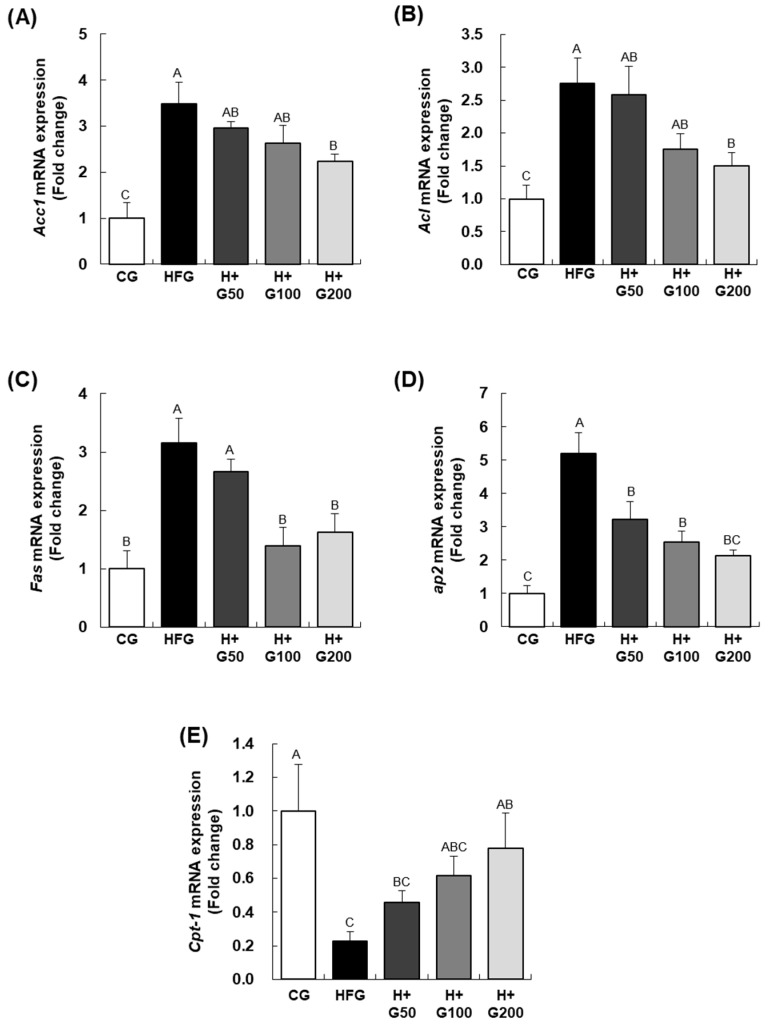
Effect of GGE03 administration on adipogenesis and lipogenesis-related gene expression in the epididymal adipose tissue of HFD-fed C57BL/6N mice. This figure highlights the modulation of adipogenesis and lipogenesis-related gene expression by GGE03 in epididymal adipose tissue of high-fat diet (HFD)-fed C57BL/6N mice. GGE03 was administered daily by oral gavage for eight weeks at doses of 50, 100, and 200 mg/kg body weight/day. Total RNA from epididymal adipose tissue was extracted, reverse-transcribed, and analyzed using real-time PCR. (**A**) Acetyl-CoA carboxylase 1 (Acc1) mRNA expression was significantly reduced in GGE03-treated groups compared to the HFD group (HFG). (**B**) ATP-citrate lyase (Acl) expression showed a dose-dependent reduction following GGE03 treatment. (**C**) Fatty acid synthase (Fas) expression was also significantly downregulated in the GGE03-treated groups. (**D**) Adipocyte protein 2 (ap2) mRNA expression was markedly lower in all GGE03-treated groups than in the HFD group. (**E**) Carnitine palmitoyltransferase-1 (Cpt-1), a gene involved in fatty acid oxidation, was significantly upregulated in GGE03-treated groups compared to the HFD group (HFG). These results suggest that GGE03 effectively suppresses adipogenesis and lipogenesis while enhancing fatty acid oxidation in a dose-dependent manner. Data are presented as mean ± standard error of the mean (SEM) (*n* = 5 per group). Statistical significance was determined by one-way ANOVA followed by Duncan’s multiple range test, and key results were confirmed with Tukey’s HSD. Different superscript letters indicate statistically significant differences among groups at *p* < 0.05. Groups sharing the same letter are not significantly different.

As shown in Figure 4, we assessed the mRNA expression of genes involved in fatty acid biosynthesis, including acetyl-CoA carboxylase 1 (*Acc1*), ATP-citrate lyase (*Acl*), fatty acid synthase (*Fas*), and adipocyte protein 2 (*ap2*). Expression levels of these genes were significantly higher in the HFG than in the CG. In the GGE03-treated groups, *Acc1* and *Acl* mRNA expression levels were significantly reduced in the H+G200 compared to the HFG group, while FAS mRNA expression was significantly reduced in both the H+G100 and H+G200. Additionally, *ap2* mRNA expression was significantly reduced in all GGE03-treated groups compared to the HFG. For *carnitine palmitoyltransferase-1* (*Cpt-1*), a gene involved in fatty acid oxidation, the HFG showed significantly lower mRNA expression compared to the CG. In contrast, all GGE03-treated groups exhibited an upward trend in *Cpt-1* expression compared to the HFG, with the increase reaching statistical significance in the H+G200. Notably, GGE03 treatment at 200 mg/kg significantly increased hepatic *Cpt-1* expression by 63.4% compared to the HFG (*p* < 0.05), suggesting enhanced fatty acid oxidation.

### 2.6. Effect of GGE03 on AMPK Phosphorylation in HFD-Induced Obese Mice

**Figure 5:**
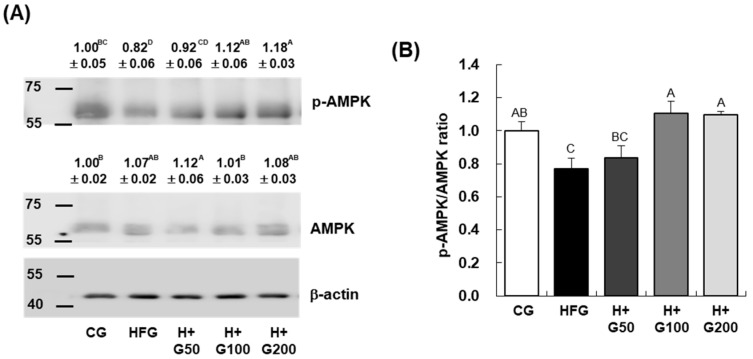
Effect of GGE03 administration on AMPK phosphorylation in the epididymal adipose tissue of HFD-fed C57BL/6N mice. This figure demonstrates the enhancement of AMPK phosphorylation (p-AMPK) in epididymal adipose tissue following GGE03 treatment in high-fat diet (HFD)-fed C57BL/6N mice. GGE03 was administered daily by oral gavage for eight weeks at doses of 50, 100, and 200 mg/kg body weight/day. Total lysates from epididymal adipose tissue were analyzed using Western blotting. (**A**) Representative Western blot images illustrate the expression levels of AMPK, p-AMPK (Thr172), and β-actin. Molecular weight markers are indicated to the left of the blots to provide reference for band sizes. (**B**) Quantification of protein band intensities normalized to β-actin shows a significant increase in the p-AMPK/AMPK ratio in GGE03-treated groups compared to the HFD group (HFG). Data are presented as mean ± standard error of the mean (SEM) (*n* = 10 per group). Statistical significance was determined by one-way ANOVA followed by Duncan’s multiple range test, and key results were confirmed with Tukey’s HSD. Different superscript letters indicate statistically significant differences among groups at *p* < 0.05. Groups sharing the same letter are not significantly different. Representative Western blot images from each group are shown; quantitative analysis was performed on normalized values across all biological replicates (*n* = 10 per group) and reflects group mean data.

As shown in Figure 5, there was no significant difference in AMPK protein expression between the HFG and CG, nor was AMPK expression affected by GGE03 administration in comparison to the HFG. However, AMPK phosphorylation (p-AMPK) was significantly reduced in the HFG compared to the CG, while administration of GGE03 at doses of 100 and 200 mg/kg BW increased p-AMPK levels compared to the HFG. Consequently, the p-AMPK/AMPK ratio, which was decreased by HFD treatment, compared to the CG, showed a significant increase in the H+G100 and H+G200.

## 3. Discussion

This study evaluated the anti-obesity effects of a standardized steamed ginger extract (GGE03), containing 1.15 mg/g of 1-dehydro-6-gingerdione, in a high-fat diet (HFD)-induced obese mouse model, focusing on body weight, adiposity, metabolic markers, and molecular pathways. While the anti-obesity effects of steamed ginger have been previously documented, including in a clinical trial by Park et al. [12], the molecular mechanisms underlying its metabolic benefits remain underexplored. The findings confirmed our hypothesis that GGE03 supplementation reduces body weight, fat mass, and serum markers of metabolic dysregulation while upregulating fatty acid oxidation pathways and AMPK phosphorylation in a dose-dependent manner. GGE03 administration resulted in significant reductions in body weight, fat mass, and weight gain efficiency compared to the HFD group. Furthermore, GGE03 improved serum metabolic markers, including glucose, triglycerides, total cholesterol, and insulin levels, while also reducing insulin resistance. At the molecular level, GGE03 downregulated key adipogenic and lipogenic genes (*C/ebp-α*, *Ppar-γ*, and *Srebp-1c*) and upregulated the fatty acid oxidation gene *Cpt-1*, particularly at higher doses. Our findings demonstrate that GGE03 exerts its effects primarily through AMPK activation, fatty acid oxidation enhancement, and suppression of lipogenesis-related pathways, rather than merely promoting weight reduction.

The reduction in body weight and fat mass observed in this study aligns with the reported effects of ginger and its bioactive components in various obesity models. Gunawan et al. [30] found that 6-gingerol ameliorated weight gain and insulin resistance in rats with metabolic syndrome by modulating adipocytokine levels. Similarly, Saravanan et al. [31] demonstrated that gingerol improved lipid profiles and reduced body weight in obese rats fed an HFD. These studies emphasize the role of ginger-derived compounds in regulating body composition and energy balance.

In our study, GGE03 treatment reduced adipocyte size and downregulated adipogenesis and lipogenesis-related genes, including *C/ebp-α*, *Ppar-γ*, and *Srebp-1c*, which are key regulators of adipocyte differentiation and lipid storage. This is consistent with findings from Gembe-Olivarez et al. [32], who reported anti-adipogenic and lipolytic effects of ginger phenols in mature adipocytes. Moreover, Deng et al. [24] highlighted the role of ginger extract and its active components, such as 6-gingerol, in promoting lipid oxidation and mitochondrial biogenesis via AMPK-PGC1α signaling. These molecular changes suggest that GGE03 effectively shifts lipid metabolism towards catabolism, reducing adiposity.

GGE03 also improved serum metabolic markers, including glucose, triglycerides, cholesterol, and insulin levels, while reducing insulin resistance and improving insulin sensitivity. De Las Heras et al. [33] observed similar hypolipidemic and insulin-sensitizing effects of ginger extract in HFD-fed rats, indicating its potential for improving metabolic health. Additionally, Wang et al. [34] demonstrated that ginger extract activated browning of white adipose tissue, contributing to enhanced energy expenditure and improved lipid profiles in obese mice. In addition to its effects on adiposity and lipid metabolism, GGE03 also improved insulin sensitivity, as evidenced by the significant reduction in HOMA-IR values in treated groups. The HOMA-IR index, calculated from fasting glucose and insulin levels, is a widely used surrogate marker for insulin resistance and plays a critical role in evaluating metabolic dysfunction associated with obesity. The observed decrease in HOMA-IR suggests that GGE03 not only modulates lipid metabolism but also contributes to improved glucose homeostasis. This finding highlights the broader metabolic impact of GGE03 and supports its potential utility in managing insulin resistance and related metabolic disorders. These findings collectively support the ability of ginger-derived compounds to modulate systemic metabolic markers.

It should be noted that daily food intake was modestly but significantly reduced (~7–8%) in all GGE03-treated groups compared with the HFD control. This reduction in caloric intake could contribute, at least in part, to the improvements observed in body weight, adiposity, and metabolic outcomes. However, the magnitude of metabolic improvements, including enhanced insulin sensitivity, reduced hepatic lipid accumulation, and increased AMPK phosphorylation, suggests that the effects of GGE03 are not solely attributable to lower energy consumption. Consistent with our findings, previous studies on steamed ginger and related phytochemicals have demonstrated metabolic benefits independent of caloric intake, implicating direct modulation of AMPK signaling and lipid metabolism [13,16]. Nevertheless, we acknowledge reduced food intake as a potential confounder, and future studies employing pair-feeding or caloric restriction controls will be essential to clearly distinguish between the pharmacological effects of GGE03 and those driven by energy intake differences.

In this study, GGE03 administration significantly reduced serum leptin levels without significantly affecting adiponectin levels. These findings align with previous research showing variability in ginger’s effects on leptin and adiponectin [33,35]. Similarly, the influence of ginger on adiponectin has been shown to depend on factors such as processing methods and dosage [30]. The absence of changes in adiponectin levels in our study suggests that GGE03 may exert a more pronounced effect on leptin regulation, potentially due to the optimized presence of 1-dehydro-6-gingerdione. This bioactive compound may preferentially target leptin pathways, thereby contributing to improved energy balance and metabolic health.

A key finding of this study is that GGE03 significantly increases AMPK phosphorylation in adipose and liver tissues. AMPK is a central regulator of energy balance, promoting catabolic pathways while inhibiting anabolic processes. The observed increase in AMPK activity was accompanied by enhanced expression of *Cpt-1*, a key gene involved in mitochondrial fatty acid oxidation. These findings suggest that GGE03 shifts energy metabolism toward lipid utilization, reducing lipid accumulation and adiposity. Unlike previous studies on ginger extracts, which focused on general weight reduction or adiposity markers, our study provides insight into the molecular mechanisms of GGE03, specifically its enhancement of AMPK activity. This finding suggests that GGE03’s anti-obesity effects could be mediated, at least in part, by stimulating AMPK-dependent pathways, further differentiating it from existing ginger extracts [24,36]. However, additional mechanistic studies are needed to confirm whether AMPK plays a direct causal role in mediating these effects. The improvements in body weight and metabolic parameters observed in vivo may result from complex systemic adaptations rather than exclusively from cell-autonomous AMPK signaling. To definitively confirm a mechanistic role, further in vitro experiments are necessary, such as treating adipocyte cell lines (e.g., 3T3-L1) with GGE03 or its key constituents to evaluate whether AMPK is directly activated and adipogenesis is inhibited under controlled conditions. These complementary studies will be essential to validate AMPK as a primary mediator of GGE03’s effects and to delineate the direct molecular actions of its bioactive compounds.

Recent studies have further reinforced the role of ginger-derived bioactives in regulating metabolic health through AMPK-dependent pathways. For instance, 6-gingerol was shown to ameliorate weight gain and insulin resistance in a metabolic syndrome model by modulating adipocytokines [30]. Similarly, Zingiber officinale extract was reported to enhance the hypolipidemic efficacy of simvastatin in obese rats, suggesting a synergistic effect on lipid metabolism [35]. Beyond ginger, other phytochemicals such as epiberberine have been demonstrated to activate AMPK and improve oxidative stress and insulin resistance in T2DM models [29]. A recent review also summarized accumulating evidence that ginger and its constituents contribute to the prevention of metabolic syndrome via antioxidant, anti-inflammatory, and AMPK-mediated mechanisms [36]. These findings align with the present study, highlighting the relevance of GGE03 as a functional food candidate targeting AMPK activation and metabolic regulation.

In addition to promoting fatty acid oxidation, GGE03 significantly downregulated key lipogenic genes, including *Srebp-1c*, *Fas*, and *Acc1*. This suppression suggests that GGE03 not only enhances lipid breakdown but also inhibits lipid synthesis, preventing further adipose expansion. Histological analysis further supports this finding, as GGE03-treated mice exhibited smaller adipocytes, indicative of reduced lipid storage and improved adipose tissue remodeling. These results aligned with previous research demonstrate that AMPK activation plays a critical role in downregulating *Srebp-1c*-mediated lipogenesis. Although the present study clearly demonstrates that GGE03 enhances AMPK phosphorylation and modulates adipogenic and lipogenic gene expression, the upstream and downstream mechanisms underlying these effects were not fully elucidated.

While our study provides strong in vivo evidence that GGE03 modulates adipose tissue remodeling and lipid metabolism, we acknowledge that the mechanistic conclusions are based solely on systemic outcomes in a high-fat diet-induced obesity model. The observed effects on adipogenic and lipogenic gene expression, adipocyte size reduction, and AMPK activation may result from both direct actions of GGE03 on adipose tissue and indirect effects mediated by hepatic lipid metabolism and circulating factors. Given the central role of the liver in regulating serum lipid and glucose levels, it is plausible that hepatocyte-mediated mechanisms contribute to the downstream remodeling of adipose tissue. In particular, the inclusion of hepatic endpoints such as *Srebp-1c*, *Fas*, *Acc*, and *Cpt-1*, along with AMPK phosphorylation, would provide valuable insights into how GGE03 influences hepatic lipid synthesis and oxidation. To more precisely delineate the cellular targets of GGE03, future studies should incorporate in vitro experiments using adipocyte (e.g., 3T3-L1) and hepatocyte (e.g., HepG2) cell models, as well as direct measurements of hepatic gene expression, protein phosphorylation, and histological changes. These investigations would help clarify whether the effects observed in vivo stem from direct action of GGE03 on adipose and hepatic tissues or from systemic metabolic regulation. Such complementary studies will be essential to fully understand the mechanisms underlying the anti-obesity and metabolic benefits of GGE03.

AMPK activation can be regulated by upstream kinases such as liver kinase B1 (LKB1) and Ca^2+^/calmodulin-dependent protein kinase kinase β (CaMKKβ), and its activation has been shown to trigger diverse downstream pathways including mitochondrial biogenesis via PGC-1α signaling and browning of white adipose tissue [24,34]. Furthermore, recent evidence suggests that phytochemicals, including ginger-derived bioactives, may exert systemic metabolic benefits through modulation of the gut microbiota, thereby influencing host energy homeostasis. While these aspects were beyond the scope of the current animal study, future work incorporating cell culture experiments, mitochondrial functional assays, and microbiome profiling will be essential to delineate the full spectrum of mechanistic actions of GGE03.

The rationale for using steamed ginger extract (GGE03) lies in its distinct phytochemical composition resulting from thermal processing. Steaming is known to transform gingerols, the primary bioactives in raw ginger, into more stable and potent derivatives such as shogaols and 1-dehydro-6-gingerdione, which have been reported to exhibit superior antioxidant, anti-inflammatory, and metabolic regulatory activities [14,15]. Previous studies have suggested that steamed ginger may exert more pronounced physiological benefits than raw or dried ginger due to these compositional changes. Park et al. (2020) [12] demonstrated in a randomized controlled clinical trial that supplementation with steamed ginger extract significantly reduced body fat and improved metabolic parameters in overweight adults, whereas comparable interventions using raw ginger extract reported more modest effects on weight and lipid profiles. These findings collectively indicate that thermal processing enhances the bioactivity of ginger by generating compounds with greater physiological potency. Consistent with this, our current HPLC and LC-MS analyses confirmed a marked elevation of 1-dehydro-6-gingerdione in GGE03 compared to raw ginger extracts. This compound has been proposed to be a key metabolic regulator through AMPK activation and insulinotropic effects, and its enrichment may underlie the enhanced anti-obesity efficacy observed in this study. However, we acknowledge that our study design did not include comparative groups receiving raw or dried ginger extracts, which represents a limitation in attributing the observed outcomes solely to the steaming process. To address this, future research should incorporate systematic comparisons among raw, dried, and steamed ginger extracts, with matched doses and standardized phytochemical characterization. Such studies should evaluate not only compositional differences but also functional outcomes in both preclinical models and clinical populations, thereby clarifying the specific contributions of each processing method to metabolic regulation and anti-obesity effects. This approach will provide definitive mechanistic evidence regarding the unique benefits of steamed ginger and inform the development of optimized ginger-based functional foods.

Nonetheless, we acknowledge that the lack of direct comparison between steamed and other ginger extracts represents a limitation of this study. While our extraction method and previous component analyses confirm the presence of bioactive compounds like 1-dehydro-6-gingerdione in GGE03, a systematic comparison of different processing methods (e.g., raw, dried, steamed) using high-performance liquid chromatography (HPLC) and functional assays would provide deeper insights into the relative efficacy of each preparation. Future studies should address this by evaluating the compositional and physiological differences between extracts and identifying which specific compounds drive the observed metabolic effects. Such comparative analysis would help clarify whether the benefits observed in this study are unique to steamed ginger or generalizable to other forms, thereby informing the development of optimized ginger-based interventions. To better evaluate the translational relevance of the doses used in this study, we estimated the human equivalent dose (HED) using the standard body surface area (BSA) normalization method recommended by the U.S. FDA. Based on this calculation, the administered doses of 50, 100, and 200 mg/kg in mice correspond to approximately 4.05, 8.1, and 16.2 mg/kg in humans, respectively, which equates to ~243, 486, and 972 mg/day for a 60 kg adult. These levels are within the range of feasible dietary or supplemental intake, considering that traditional consumption of ginger often reaches several grams per day in various food and herbal preparations. Furthermore, a recent randomized controlled trial using steamed ginger ethanolic extract reported significant anti-obesity effects at comparable daily doses without notable adverse effects [12], supporting the safety and practicality of such intake levels. Collectively, these findings suggest that the effective doses of GGE03 identified in our animal model may be reasonably translated into human dietary supplementation, thereby reinforcing its potential as a functional food candidate for obesity management.

Interestingly, in this study GGE03 supplementation significantly reduced circulating leptin levels, whereas adiponectin levels remained unchanged. This selective effect may be attributable to several factors. First, the phytochemical composition of GGE03 differs from that of raw or dried ginger extracts, as steaming enriches 1-dehydro-6-gingerdione while reducing gingerols, potentially shifting the regulatory balance toward leptin pathways rather than adiponectin modulation [13,16]. Second, treatment duration and cumulative exposure may play a role, since studies reporting increased adiponectin often employed longer interventions or higher cumulative doses of ginger preparations [22,33]. Third, tissue-specific actions of ginger bioactives may also contribute; previous reports suggest differential effects of ginger constituents on hepatic, adipose, and pancreatic tissues, with adiponectin responses being more variable than leptin regulation [24,34]. Taken together, these findings suggest that GGE03 may exert more pronounced effects on leptin-mediated energy balance, while modulation of adiponectin may require different treatment parameters. Future studies with extended treatment periods and direct tissue-level assessments are warranted to clarify these adipokine-specific responses. The absence of significant effects on adiponectin levels despite reduced leptin levels in GGE03-treated groups is also noteworthy. While ginger’s effects on adiponectin have been reported inconsistently across studies, our results suggest that GGE03 may predominantly influence leptin pathways, which could impact energy intake regulation. This selective effect on leptin may be due to the higher content of 1-dehydro-6-gingerdione in GGE03, indicating that the bioactive profile of steamed ginger could affect its hormonal regulation.

While much of the research on ginger’s anti-obesity effects has focused on raw ginger and its constituents, such as gingerol [37,38], shogaol [17], and gingerenone A [39], our study demonstrates the potential of a standardized steamed ginger extract like GGE03. The presence of 1-dehydro-6-gingerdione in GGE03 may provide unique metabolic benefits, as steaming alters the chemical composition of ginger and enhances the bioavailability of specific bioactives [40,41]. Furthermore, systematic reviews and meta-analyses, such as those by Ebrahimzadeh Attari et al. [42] and Zhu et al. [43], have emphasized ginger’s multifaceted effects on obesity and metabolic syndrome, underscoring the need for standardized extracts to maximize efficacy. Although our study was conducted in an animal model, the findings hold translational significance. The observed metabolic effects of GGE03 align with known AMPK-activating agents used in metabolic disease management, such as metformin. However, the high doses used in our study (50–200 mg/kg) necessitate further pharmacokinetic evaluations to determine clinically relevant dosing in humans. Future research should include pharmacokinetic and pharmacodynamic studies, as well as pilot clinical trials, to assess the safety and efficacy of GGE03 in human subjects.

Despite its strengths, this study has certain limitations. First, only male C57BL/6N mice were used, which limits the generalizability of the findings to female subjects. Given the known sex differences in metabolic regulation and adipose tissue biology, future studies should include both sexes to provide a more comprehensive understanding of GGE03’s effects. Second, although food intake was monitored and recorded, a modest but significant reduction in intake (~7–8%) was observed in GGE03-treated groups. Without a pair-fed control group, it is difficult to fully disentangle whether the improvements in body weight and metabolic parameters were due to the direct pharmacological actions of GGE03 or simply to reduced caloric intake. Future experiments should include pair-fed designs to rigorously address this potential confounder. Third, the diet used in this study contained 60% kcal from fat, which is a well-established and reproducible model for rapidly inducing obesity and insulin resistance in rodents. However, this model reflects only one dimension of human obesity, which is also strongly associated with high consumption of sugars and fructose. Thus, while the 60% HFD provides a robust platform to evaluate the metabolic effects of GGE03, it does not fully capture the complexity of human dietary patterns. Future studies should incorporate more representative diet models, such as high-fat/high-sucrose or high-fat/high-fructose regimens, to better mimic human obesity and to validate the efficacy of GGE03 under conditions of mixed nutrient excess. Fourth, the reliance on a single animal model (C57BL/6N mice) may not fully capture the metabolic diversity observed in human obesity. Although we did not perform a separate toxicity evaluation in this study, previous clinical and preclinical reports have demonstrated the safety of steamed ginger extract at doses comparable to or higher than those used herein [12]. In this study, we directly characterized the exact batch of GGE03 used for the animal experiments through HPLC analysis, confirming the presence and quantity of key bioactive compounds, including 1-dehydro-6-gingerdione, 6-gingerol, and 6-shogaol. The concentration of 1-dehydro-6-gingerdione was determined to be 11.77 μg/mL, validating that the biological effects observed in vivo were derived from a chemically standardized preparation. While this strengthens the reproducibility of our findings, it remains unclear whether other minor compounds within the extract contribute synergistically to the observed anti-obesity effects. Further research should explore the individual and combined actions of these constituents to identify specific contributors to GGE03’s metabolic activity. Fifth, although GGE03 administration improved obesity-related outcomes, the observed effects were not strictly proportional across the tested doses (50, 100, and 200 mg/kg). This lack of a clear dose–response relationship suggests that even the lowest dose may have been sufficient to elicit near-maximal effects. Such a pattern is consistent with the presence of a ceiling effect, in which increasing the dose beyond a certain threshold does not confer additional benefits. These results raise the possibility that the lower doses of GGE03 may already represent a minimal effective dose, beyond which higher doses provide no further improvement. Similar non-linear dose–response curves have been reported for other phytochemicals, where factors such as bioavailability limitations, receptor binding saturation, or compensatory metabolic adaptations constrain the magnitude of physiological effects. In addition, inter-animal variability and the limited sample size in this study may have obscured more subtle differences between dose groups. Future research should incorporate broader dose ranges, pharmacokinetic evaluations, and longer intervention periods to clarify the true dose–response relationship and determine the minimal effective dose of GGE03. These studies will be essential to define the therapeutic window, optimize dosing strategies, and better understand the pharmacological and metabolic actions of GGE03 in both preclinical models and potential clinical applications. Finally, while AMPK activation was identified as a key molecular mechanism in adipose tissue, this study did not examine upstream regulators or downstream metabolic pathways in other tissues such as the liver, skeletal muscle, or gut microbiota. Future investigations should incorporate broader tissue analyses and include in vitro studies using adipocyte and hepatocyte cell lines to determine whether GGE03 directly activates AMPK in a cell-autonomous manner. Moreover, evaluating lower, clinically relevant doses and long-term administration will be essential to determine the feasibility of GGE03 as a functional food or therapeutic candidate for obesity and metabolic syndrome.

## 4. Materials and Methods

All experimental procedures were carefully standardized, validated, and performed under controlled conditions to ensure reproducibility and scientific rigor.

### 4.1. Preparation and Characterization of GGE03

GGE03 was generously provided by Genencell Co., Ltd. (Yongin, Republic of Korea) and prepared following a standardized procedure [44,45]. Briefly, fresh ginger was washed three times with water, steamed at 97 °C for 2 h, and dried at 50 °C for 30 h. The steamed ginger was extracted with a fifteen-fold volume of 70% ethanol (*v*/*v*) at 85 °C for 15 h. After extraction, the solution was filtered and concentrated via vacuum evaporation, and the concentrate was spray-dried to obtain a final powdered product containing less than 5% moisture.

To ensure reproducibility and to directly characterize the exact batch used in the animal study, high-performance liquid chromatography (HPLC) analysis was performed using a Waters e2695 system equipped with a photodiode array detector (Waters Co., Milford, MA, USA). Chromatographic separation was achieved on a Cadenza C18 column (250 × 4.6 mm, 3 µm; Imtakt, Portland, OR, USA) maintained at 25 °C. The mobile phase consisted of solvent A (0.1% acetic acid in distilled water) and solvent B (acetonitrile) with a flow rate of 0.4 mL/min under the following linear gradient conditions (A/B, *v*/*v*): 0–5 min, 70/30; 10–13 min, 45/55; 20–23 min, 20/80; 30–60 min, 0/100, followed by re-equilibration to 70/30 at 62–80 min. The injection volume was 10 µL, and the detection wavelength was 370 nm. HPLC-grade acetonitrile and distilled water (Honeywell International Inc., Charlotte, NC, USA) were used for all analyses.

**Figure 6:**
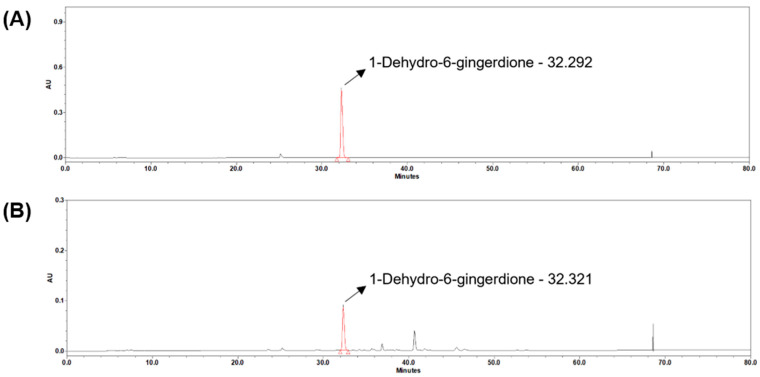
Representative chromatograms obtained by high-performance liquid chromatography with photodiode array detection (HPLC-PDA). (**A**) 1-dehydro-6-gingerdione standard solution at a concentration of 29.94 μg/mL. (**B**) Steamed ginger extract (GGE03) used in the animal experiments, containing 1-dehydro-6-gingerdione at a concentration of 11.77 μg/mL. Peaks confirm the characteristic phytochemical profile of GGE03, validating the exact batch used in this study.

The resulting chromatogram confirmed the presence of key marker compounds, including 1-dehydro-6-gingerdione, 6-gingerol, and 6-shogaol, with 1-dehydro-6-gingerdione quantified at 11.77 µg/mL in the tested batch. These results (Figure 6) verify the characteristic phytochemical profile of steamed ginger and ensure that the extract used in this study was chemically validated and standardized.

### 4.2. Ethical Statements and Animals

All animal experimental protocols in this study were approved by the Institutional Animal Care and Use Committee of Hallym University (approval number: Hallym 2024-4, approval date: 7 May 2024). Animal care and experimental procedures adhered to the institutional guidelines for the care and use of laboratory animals and complied with the National Animal Welfare Law of the Republic of Korea.

Four-week-old male C57BL/6N mice were obtained from Doo Yeol Biotech Co., Ltd. (Seoul, Republic of Korea). The mice were housed in the animal facility at Hallym University under controlled environmental conditions (23 ± 3 °C, 50 ± 10% relative humidity, and a 12 h light/dark cycle). To minimize potential confounders, animals were housed in identical conditions within the same room, with cages randomly positioned on racks to eliminate location effects. Food and water were provided ad libitum, and all treatments were administered at the same time each day to reduce variability due to circadian rhythms. Measurements, including body weight and food intake, were taken in a consistent order for all groups to avoid systematic bias. Throughout the experimental period, animals were closely monitored for general health indicators, including body weight, food intake, and behavioral abnormalities, and no adverse effects related to GGE03 administration were observed. No additional confounders were identified or explicitly controlled beyond these measures.

### 4.3. Experimental Design and Treatment

After a one-week acclimatization period in a controlled environment with free access to standard chow and water, the C57BL/6N mice were randomly assigned to five groups (*n* = 10 per group) as follows: (i) control diet (CD) group (CG), (ii) high-fat diet (HFD) group (HFG), (iii) HFD + 50 mg/kg body weight (BW)/day GGE03-treated group (H+G50), (iv) HFD + 100 mg/kg BW/day GGE03-treated group (H+G100), and (v) HFD + 200 mg/kg BW/day GGE03-treated group (H+G200). GGE03 was prepared fresh daily and administered orally using a gavage needle once per day at the same time each morning for eight consecutive weeks. Randomization was conducted using a computer-generated randomization sequence to ensure unbiased allocation of animals to experimental groups.

The CG group received a control diet (CD) with 10% of calories from fat, 20% protein, and 70% carbohydrates (Catalog No. D12450B, Research Diets, Inc., New Brunswick, NJ, USA; see Table 3). In contrast, the remaining four groups were fed a high-fat diet (HFD) consisting of 60% fat, 20% protein, and 20% carbohydrates (Catalog No. D12492, Research Diets, Inc.; see Table 4). All animals had unrestricted access to their respective diets and water for a total of eight weeks. GGE03, dissolved in sterile water, was administered daily via oral gavage for eight weeks. The CG and HFG groups received an equivalent volume of sterile water by oral gavage. Food intake was recorded daily, and body weight was measured weekly throughout the GGE03 treatment period.

During the experimental period, body weight and food intake were measured weekly to monitor growth and dietary consumption. At the end of the 8-week treatment period, the mice were fasted for 16 h overnight to standardize metabolic conditions before sample collection. The mice were then anesthetized using tribromoethanol diluted in tertiary amyl alcohol (dose: 250 mg/kg BW, intraperitoneally) to minimize stress and ensure humane treatment during blood collection. Blood samples were collected from the orbital vein using a capillary tube, and serum was separated by centrifugation at 1500× *g* for 20 min at 4 °C.

The primary outcome measure for this study was body weight, which was used to determine the sample size. Secondary outcome measures included fat mass percentage, white adipose tissue (WAT) weight, serum biochemical markers (glucose, lipids, insulin, leptin, and adiponectin), and molecular markers such as AMPK phosphorylation and gene expression related to lipid metabolism. Following blood collection, the mice were euthanized by cervical dislocation. WAT from four regions (epididymal, retroperitoneal, mesenteric, and inguinal) and the liver were promptly excised, rinsed with phosphate-buffered saline to remove blood, blotted dry, and weighed.

### 4.4. Body Composition Analysis

One day before the conclusion of the experiment, fat mass percentages were assessed using dual-energy X-ray absorptiometry (DEXA) with a PIXImus™ scanner (GE Lunar, Madison, WI, USA).

### 4.5. Serum Biochemical Analysis

Serum concentrations of glucose, triglycerides, total cholesterol, low-density lipoprotein (LDL) cholesterol, and high-density lipoprotein (HDL) cholesterol were analyzed using an automated biochemical analyzer (Indiko Plus, Thermo Fisher Scientific, Vantaa, Finland). Serum leptin (No. EZML-82K, Millipore, Billerica, MA, USA), adiponectin (No. MRP300, R&D Systems, Minneapolis, MN, USA), and insulin (No. EZRMI-13K, Millipore) levels were quantified using specific enzyme-linked immunosorbent assay (ELISA) kits, following the manufacturer’s instructions. Insulin resistance was calculated using the homeostasis model assessment of insulin resistance (HOMA-IR) with the following formula: HOMA-IR = [fasting glucose (mg/dL) × fasting insulin (µU/mL)]/405 [46]. To assess insulin sensitivity, the quantitative insulin sensitivity check index (QUICKI) was used and calculated as follows: QUICKI = 1/[log(fasting glucose [mg/dL]) + log(fasting insulin [µU/mL])] [47].

### 4.6. Histological Analysis

Epididymal adipose tissues were fixed in 4% paraformaldehyde, embedded in paraffin, and sectioned at a thickness of 5 µm. The sections underwent deparaffinization, followed by rehydration through a graded ethanol series, and were subsequently stained with hematoxylin and eosin (H&E) for histological evaluation. Microscopic examination was performed using a light microscope (AxioImager, Carl Zeiss, Jena, Germany), and representative images were captured. Adipocyte size was quantified from H&E-stained epididymal adipose tissue sections using the AxioVision Imaging System (Carl Zeiss) at 200× magnification. For each animal, five independent tissue sections were prepared, and at least three randomly selected, non-overlapping microscopic fields per section were analyzed to reduce sampling bias. All measurements were performed in a blinded manner to ensure objectivity and minimize observer bias.

### 4.7. Western Blot Analysis

Epididymal adipose tissue samples were homogenized using established protocols as previously reported [48]. Western blotting was conducted following standard procedures described in earlier studies [49]. Primary antibodies specific for AMP-activated protein kinase α (AMPKα, Cat. No. 2532), phosphorylated AMPKα at threonine 172 (p-AMPKα, Cat. No. 2535), and β-actin (Cat. No. 3700) were purchased from Cell Signaling Technology (Beverly, MA, USA). Immunoreactive bands were detected using the Luminata™ Forte Western HRP Substrate (Cat. No. WBLUF0500, Millipore), and the signal intensities were measured with the ImageQuant™ LAS 500 imaging system (GE Healthcare Bio-Sciences AB, Uppsala, Sweden). The expression of target proteins was normalized to β-actin levels.

### 4.8. Quantitative Reverse Transcription-Polymerase Chain Reaction (RT-PCR)

Total RNA from epididymal adipose tissue was extracted using TRIzol reagent (No. 1596018, Thermo Fisher Scientific, Waltham, MA, USA). Real-time RT-PCR was performed using a HyperScript™ RT Master Mix kit (No. 601710, GeneAll Biotechnology, Seoul, Republic of Korea), a QuantiNova SYBR Green PCR kit (No. 208056, Qiagen, Valencia, CA, USA), and a Rotor-Gene 3000 instrument (Corbett Research, Mortlake, Australia), following a previously described protocol [50]. The primer sequences utilized for quantitative PCR are provided in Table 5. Gene expression data were processed using Rotor-Gene 6000 Series System Software (version 6, Corbett Research), and the expression levels of each target gene were normalized to that of the internal reference gene, glyceraldehyde-3-phosphate dehydrogenase (*Gapdh*).

### 4.9. Statistical Analysis

All data are presented as the mean ± standard error of the mean (SEM) for each experimental group. Statistical analyses were performed using the Statistical Analysis System (SAS), Windows version 9.4 (SAS Institute, Cary, NC, USA). Differences among groups were evaluated using one-way analysis of variance (ANOVA). Post hoc comparisons were initially conducted using Duncan’s multiple range test, which is widely used in nutritional and biomedical studies with balanced sample sizes. To confirm the robustness of our findings, key datasets were re-analyzed using Tukey’s honestly significant difference (HSD) test, a more conservative post hoc method. Both tests yielded consistent patterns of statistical significance, supporting the reliability of the reported results. A *p*-value of less than 0.05 was considered statistically significant. Sample sizes for each group were *n* = 10 unless otherwise specified, and these details are also noted in each figure legend for clarity.

## 5. Conclusions

This study demonstrated that GGE03, a standardized steamed ginger ethanolic extract, exerts potent anti-obesity effects by promoting fatty acid oxidation, suppressing lipogenesis, and enhancing AMPK phosphorylation in a high-fat diet-induced obesity model. Importantly, our findings reveal that GGE03 influences key metabolic pathways associated with energy homeostasis and adipocyte remodeling, providing novel mechanistic insights beyond previously reported clinical outcomes. These results support the therapeutic potential of GGE03 as a functional food ingredient for obesity management.

However, while our data indicate that the beneficial effects of GGE03 are associated with AMPK activation and lipid metabolism regulation, they do not establish direct causality. Further mechanistic studies are required to confirm whether these effects are directly mediated by AMPK signaling and to delineate the contributions of individual bioactive compounds within GGE03. Future studies should also validate these findings in clinical populations and assess the long-term safety and efficacy of GGE03 through well-designed clinical trials.

## Figures and Tables

**Table 1:** Effect of GGE03 administration on body weight gain, fat mass percentages, and food intake in HFD-induced obese C57BL/6N mice.

	CG	HFG	H+G50	H+G100	H+G200
Initial body weight (g) ^(1)^	20.4 ± 0.2	20.8 ± 0.2	20.8 ± 0.2	20.5 ± 0.2	20.4 ± 0.2
Final body weight (g)	29.7 ± 0.6 ^c^	39.0 ± 0.4 ^a^	34.6 ± 0.8 ^b^	34.0 ± 0.5 ^b^	33.6 ± 0.4 ^b^
Body weight gain (g)	9.3 ± 0.4 ^c^	18.1 ± 0.3 ^a^	13.8 ± 0.6 ^b^	13.4 ± 0.3 ^b^	13.4 ± 0.2 ^b^
Food intake (g/day)	2.65 ± 0.02 ^a^	2.24 ± 0.01 ^b^	2.08 ± 0.02 ^c^	2.09 ± 0.02 ^c^	2.07 ± 0.02 ^c^
Food efficiency ratio ^(2)^	0.064 ± 0.003 ^c^	0.148 ± 0.003 ^a^	0.121 ± 0.005 ^b^	0.117 ± 0.003 ^b^	0.117 ± 0.002 ^b^

^(1)^ This table summarizes the impact of GGE03 administration on body weight gain, fat mass percentages, and food intake in C57BL/6N mice fed a high-fat diet (HFD). ^(2)^ The food efficiency ratio was calculated as weight gain divided by food intake, providing an indicator of the conversion of consumed calories into body weight. Values are expressed as the mean ± standard error of the mean (SEM) (*n* = 10 per group). Statistical significance was determined by one-way ANOVA followed by Duncan’s multiple range test, and key results were confirmed with Tukey’s HSD. Different superscript letters indicate statistically significant differences among groups at *p* < 0.05. Groups sharing the same letter are not significantly different.

**Table 2:** Effect of GGE03 administration on serum glucose, lipids, insulin, leptin, and adiponectin levels in HFD-induced obese C57BL/6N mice.

	CG	HFG	H+G50	H+G100	H+G200
Glucose (mg/dL) ^(1)^	175.0 ± 8.8 ^c^	220.2 ± 8.2 ^b^	242.9 ± 6.4 ^a^	172.9 ± 4.3 ^c^	169.6 ± 5.9 ^c^
Triglyceride (mg/dL)	63.3 ± 4.9 ^b^	82.9 ± 4.2 ^a^	79.9 ± 3.6 ^a^	62.1 ± 4.5 ^b^	57.2 ± 2.9 ^b^
Total cholesterol (mg/dL)	151.5 ± 5.1 ^b^	183.5 ± 5.3 ^a^	169.8 ± 3.6 ^ab^	163.4 ± 4.9 ^b^	159.9 ± 9.5 ^b^
Insulin (ng/mL)	1.90 ± 0.23 ^c^	4.81 ± 0.28 ^a^	3.67 ± 0.22 ^b^	3.33 ± 0.25 ^b^	3.36 ± 0.37 ^b^
HOMA-IR ^(2)^	19.3 ± 2.2 ^d^	62.3 ± 3.6 ^a^	52.7 ± 3.1 ^b^	34.2 ± 2.9 ^c^	34.3 ± 4.2 ^c^
QUICKI ^(3)^	0.259 ± 0.004 ^a^	0.228 ± 0.001 ^c^	0.232 ± 0.002 ^c^	0.242 ± 0.002 ^b^	0.244 ± 0.004 ^b^
Leptin (ng/mL)	13.8 ± 0.4 ^c^	31.5 ± 1.1 ^a^	25.2 ± 1.8 ^b^	23.1 ± 1.2 ^b^	22.8 ± 1.1 ^b^
Adiponectin (μg/mL)	9.71 ± 0.17 ^a^	8.87 ± 0.17 ^b^	8.98 ± 0.18 ^b^	8.80 ± 0.15 ^b^	8.81 ± 0.13 ^b^

^(1)^ This table summarizes the effects of GGE03 administration on key metabolic markers, including serum glucose, lipids, insulin, leptin, and adiponectin levels in C57BL/6N mice fed a high-fat diet (HFD). ^(2)^ Homeostasis model assessment of insulin resistance (HOMA-IR) was calculated using the formula: [fasting glucose (mg/dL) × fasting insulin (mU/L)/405]. ^(3)^ Quantitative insulin sensitivity check index (QUICKI) was calculated using the formula: 1/[log fasting glucose (mg/dL) + log fasting insulin (mU/L)]. Values are expressed as the mean ± standard error of the mean (SEM) (*n* = 10 per group). Statistical significance was determined by one-way ANOVA followed by Duncan’s multiple range test, and key results were confirmed with Tukey’s HSD. Different superscript letters indicate statistically significant differences among groups at *p* < 0.05. Groups sharing the same letter are not significantly different.

**Table 3:** Rodent diet with 10 kcal% fat.

Class Description	Ingredients ^(1)^	Grams ^(2)^
Protein	Casein, Lactic, 30 Mesh	200.00 g
Protein	Cystine, L	3.00 g
Carbohydrate	Sucrose, Fine Granulated	354.00 g
Carbohydrate	Starch, Corn	315.00 g
Carbohydrate	Lodex 10	35.00 g
Fiber	Solka Floc, FCC200	50.00 g
Fat	Soybean Oil, USP	25.00 g
Fat	Lard	20.00 g
Mineral	S10026B	50.00 g
Vitamin	Choline Bitartrate	2.00 g
Vitamin	V10001C	1.00 g
Dye	Dye, Yellow FD&C #5, Alum. Lake 35–42%	0.05 g
	Total:	1055.05 g

^(1)^ This table details the formulation of the control diet (CD) used in the study, containing 10% of calories from fat, 20% from protein, and 70% from carbohydrates. ^(2)^ The diet was prepared with precise ingredient weights to achieve a total of 1055.05 g per batch. Ingredients include protein sources such as casein and cystine, carbohydrate sources like sucrose and starch, and fat sources including soybean oil and lard. Fiber, minerals, vitamins, and dye were added to ensure a nutritionally complete diet. Nutritional content was calculated based on ingredient composition, and all diets were prepared under standardized conditions to ensure consistency. No significant variation in nutrient composition or caloric density was observed between batches. Means are expressed as grams per total diet weight.

**Table 4:** Rodent diet with 60 kcal% fat.

Class Description	Ingredients ^(1)^	Grams ^(2)^
Protein	Casein, Lactic, 30 Mesh	200.00 g
Protein	Cystine, L	3.00 g
Carbohydrate	Lodex 10	125.00 g
Carbohydrate	Sucrose, Fine Granulated	72.80 g
Fiber	Solka Floc, FCC200	50.00 g
Fat	Lard	245.00 g
Fat	Soybean Oil, USP	25.00 g
Mineral	S10026B	50.00 g
Vitamin	Choline Bitartrate	2.00 g
Vitamin	V10001C	1.00 g
Dye	Dye, Blue FD&C #1, Alum. Lake 35–42%	0.05 g
	Total:	773.85 g

^(1)^ This table presents the formulation of the high-fat diet (HFD) used in the study, containing 60% of calories from fat, 20% from protein, and 20% from carbohydrates. ^(2)^ The diet was prepared with a total weight of 773.85 g per batch. Protein sources include casein and cystine, while carbohydrate sources consist of Lodex 10 and sucrose. Fat content is derived from lard and soybean oil. Fiber, minerals, vitamins, and dye were incorporated to ensure a balanced and complete diet suitable for experimental use. Nutritional content was standardized across all batches to maintain consistency in experimental conditions. Ingredient weights were measured to ensure accuracy, and the caloric density was calculated based on ingredient composition. No significant variation was detected in macronutrient proportions or overall caloric density between prepared batches.

**Table 5:** Primer sequences used in this study.

Target Gene ^(1)^	Forward Primer (5′-3′)	Reverse Primer (5′-3′)
*Acc1* ^(2)^	GGAGATGTACGCTGACCGAGAA	ACCCGACGCATGGTTTTCA
*Acl*	TGGATGCCACAGCTGACTAC	GGTTCAGCAAGGTCAGCTTC
*ap2*	GGATTTGGTCACCATCCGGT	TTCACCTTCCTGTCGTCTGC
*C/ebp-* *α*	TGGACAAGAACAGCAACGAGTAC	GCAGTTGCCCATGGCCTTGAC
*Cpt-1*	GTGCTGGAGGTGGCTTTGGT	TGCTTGACGGATGTGGTTCC
*Fas*	AGGGGTCGACCTGGTCCTCA	GCCATGCCCAGAGGGTGGTT
*Ppar-γ*	CAAAACACCAGTGTGAATTA	ACCATGGTAATTTCTTGTGA
*Srebp-1c*	CACTTCTGGAGACATCGCAAAC	ATGGTAGACAACAGCCGCATC
*Gapdh*	TGGGTGTGAACCATGAGAAG	GCTAAGCAGTTGGTGGTGC

^(1)^ This table lists the forward and reverse primer sequences used for quantitative reverse transcription-polymerase chain reaction (RT-PCR) analysis to measure gene expression levels in this study. Each target gene is accompanied by its respective primer sequences, which were designed for optimal specificity and efficiency. All primers were validated for amplification efficiency and specificity under standardized PCR conditions. ^(2)^ Gene abbreviations and their corresponding full names are as follows: *Acc1*: Acetyl-CoA carboxylase 1, *Acl*: ATP-citrate lyase, *ap2*: Adipocyte protein 2, *C/ebp-α*: CCAAT/enhancer binding protein-α, *Cpt-1*: Carnitine palmitoyltransferase-1, *Fas*: Fatty acid synthase, *Ppar-γ*: Peroxisome proliferator-activated receptor-γ, *Srebp-1c*: Sterol regulatory element-binding protein-1c, *Gapdh*: Glyceraldehyde-3-phosphate dehydrogenase (used as a housekeeping gene for normalization).

## Data Availability

The data used to support of this study are included with the article.
